# Recurrent Myocardial Infarction Despite Normal C-reactive Protein in a Patient with Behcet’s Disease and Compound Heterozygous Methylenetetrahydrofolate Reductase (MTHFR) Mutations (C677T and A1298C)

**DOI:** 10.7759/cureus.5344

**Published:** 2019-08-08

**Authors:** Muhammad Hamza Saad Shaukat, Aixa Toledo-Garcia, Mikhail Torosoff

**Affiliations:** 1 Internal Medicine, Albany Medical College, Albany, USA; 2 Rheumatology, Center for Rheumaology, New York, USA; 3 Cardiology, Albany Medical Center Hospital, Albany, USA

**Keywords:** behcets, recurrent myocardial infarction, mthfr mutations

## Abstract

A 39-year-old diabetic female with Behcet’s disease presented with acute inferior wall myocardial infarction and underwent successful angioplasty of the occluded circumflex artery with a bare-metal stent (balancing increased the bleeding risk with Behcet's). Other coronary vessels were free of obstructive atherosclerosis. Optimal coronary artery disease (CAD) therapy was commenced, and Behcet’s disease treatment was intensified with the normalization of C-reactive protein. Two years later, she presented with an acute left anterior descending artery occlusion that was managed with a drug-eluting stent this time. There was no evidence of diffuse atherosclerosis on coronary angiogram or coronary calcifications on the chest computed tomography (CT) scan. Compound heterozygous methylenetetrahydrofolate reductase (MTHFR) mutations (C677T and A1298C) and high-normal plasma homocysteine were detected. With the long-term continuation of dual anti-platelet, lipid-lowering, immunosuppressive, and folic-acid therapies, she did not have cardiac events during the three-year follow-up. This is the first report of recurrent thrombotic acute coronary syndrome (ACS) in a patient with diabetes, compound heterozygous MTHFR mutations, Behcet’s disease with normal C-reactive protein (CRP), and no evidence of diffuse coronary artery disease.

## Introduction

This is a case of recurrent thrombotic myocardial infarction in a 41-year-old non-smoking woman with diabetes, Behcet’s disease, and methylenetetrahydrofolate reductase (MTHFR) mutations.

Diabetes mellitus is a well-established risk factor for atherosclerosis, which commonly presents with multivessel obstructive coronary artery disease (CAD) [1]. Despite a history of diabetes, the presented patient did not have diffuse coronary artery disease, as attested by two coronary angiograms, and no coronary calcifications were noted on the chest computed tomography (CT) scan.

Acute coronary syndrome and accelerated atherosclerosis are uncommon in patients with well-controlled Behcet’s disease [2-3]. Immunosuppression is the mainstay for the prevention of coronary thrombosis in Behcet’s disease, in addition to guideline-directed medical therapy for coronary artery disease (CAD) balanced against the increased risk of bleeding [4].

MTHFR mutations (C677T and A1298C) have been linked to an elevated cardiovascular risk in patients with rheumatoid arthritis, deep-venous thrombosis, and ischemic stroke [5-6]. Yet, there is no consensus on whether the isolated presence of these mutations is associated with acute coronary syndromes.

The influence of compound heterozygous MTHFR mutations on cardiovascular risk in patients with Behcet’s disease and diabetes is uncertain. The thrombotic events noted in this case are not typically seen in patients with diabetes or Behcet’s disease with normal inflammatory markers. Therefore, it is plausible to infer that MTHFR mutations contributed to recurrent thrombotic coronary events. This case highlights the importance of a thorough investigation for pro-thrombotic conditions in a relatively young patient with a systemic autoimmune disease presenting with recurrent acute coronary syndrome.

## Case presentation

**Figure 1:**
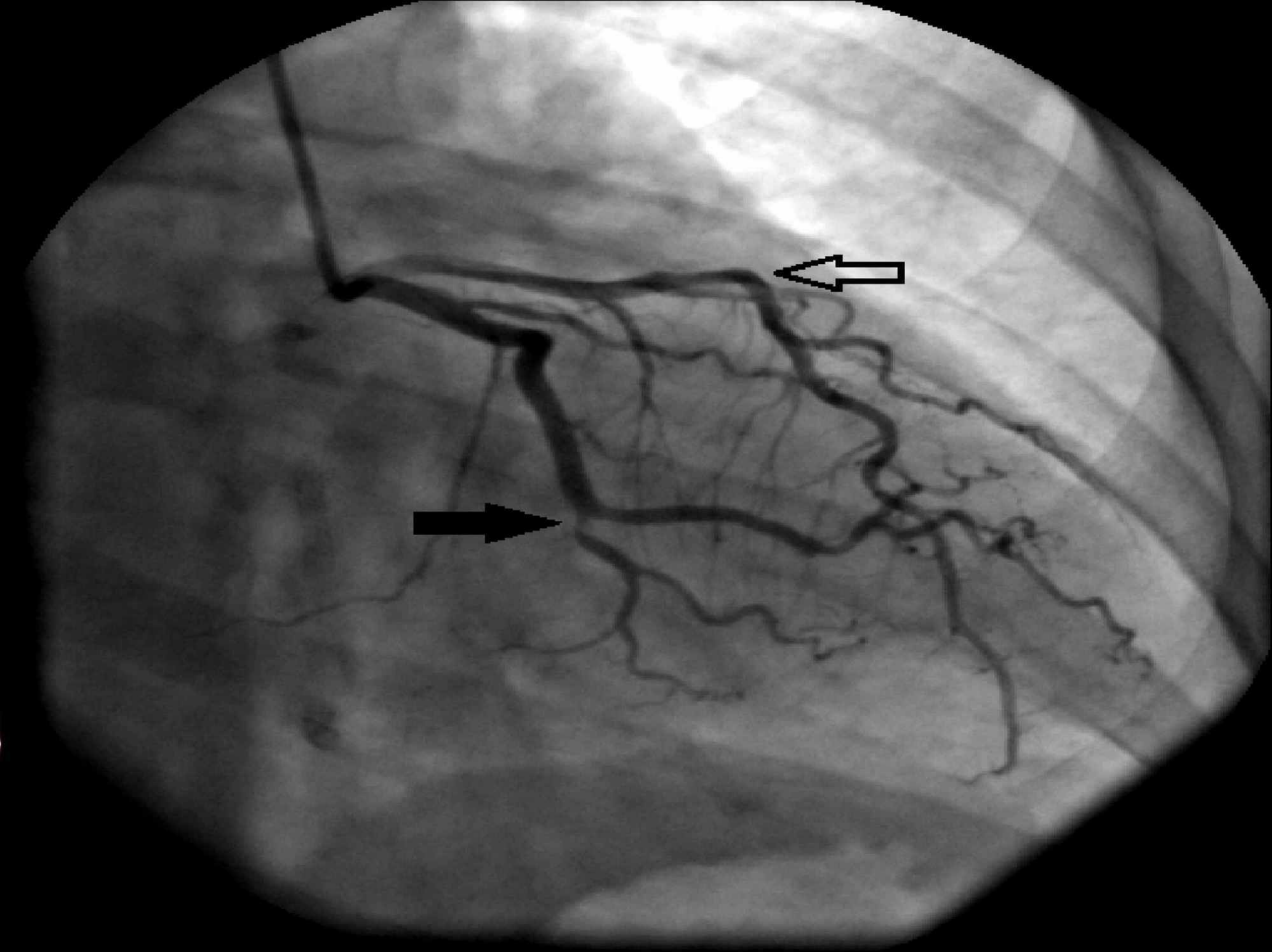
Right anterior-oblique caudal view on coronary angiogram Occluded circumflex artery (closed arrow) and patent left anterior descending artery (open arrow) on coronary angiogram at initial presentation. Besides the circumflex artery occlusion, no significant obstructive disease was present.

A 39-year-old type 1 diabetic Caucasian female, non-smoker, HLA-B51 positive, with Behcet’s disease, presented with acute inferior ST-elevation myocardial infarction and underwent a bare-metal stent percutaneous coronary intervention (PCI) to the occluded circumflex artery (Figure 1). No other obstructive coronary lesions were detected. There was no familial or personal history of premature coronary artery disease, ischemic stroke, deep vein thrombosis (DVT), pulmonary embolism, or miscarriage. Mild dyslipidemia was noted, with total cholesterol of 217 mg/d, low-density lipoprotein (LDL) of 148 mg/dL, and high-density lipoprotein (HDL) of 58 mg/dL. Optimal medical therapy for coronary artery disease (CAD) was commenced, and the treatment of Behcet’s disease was intensified. Since the patient had limited response to hydroxychloroquine and intolerance to colchicine, methotrexate was initiated with the resolution of the patient’s mucocutaneous ulcers, sinusitis, and pseudo-folliculitis and the normalization of elevated C-reactive protein (CRP). The patient was maintained on optimal medical therapy for CAD, including dual anti-platelets for six months.

**Figure 2:**
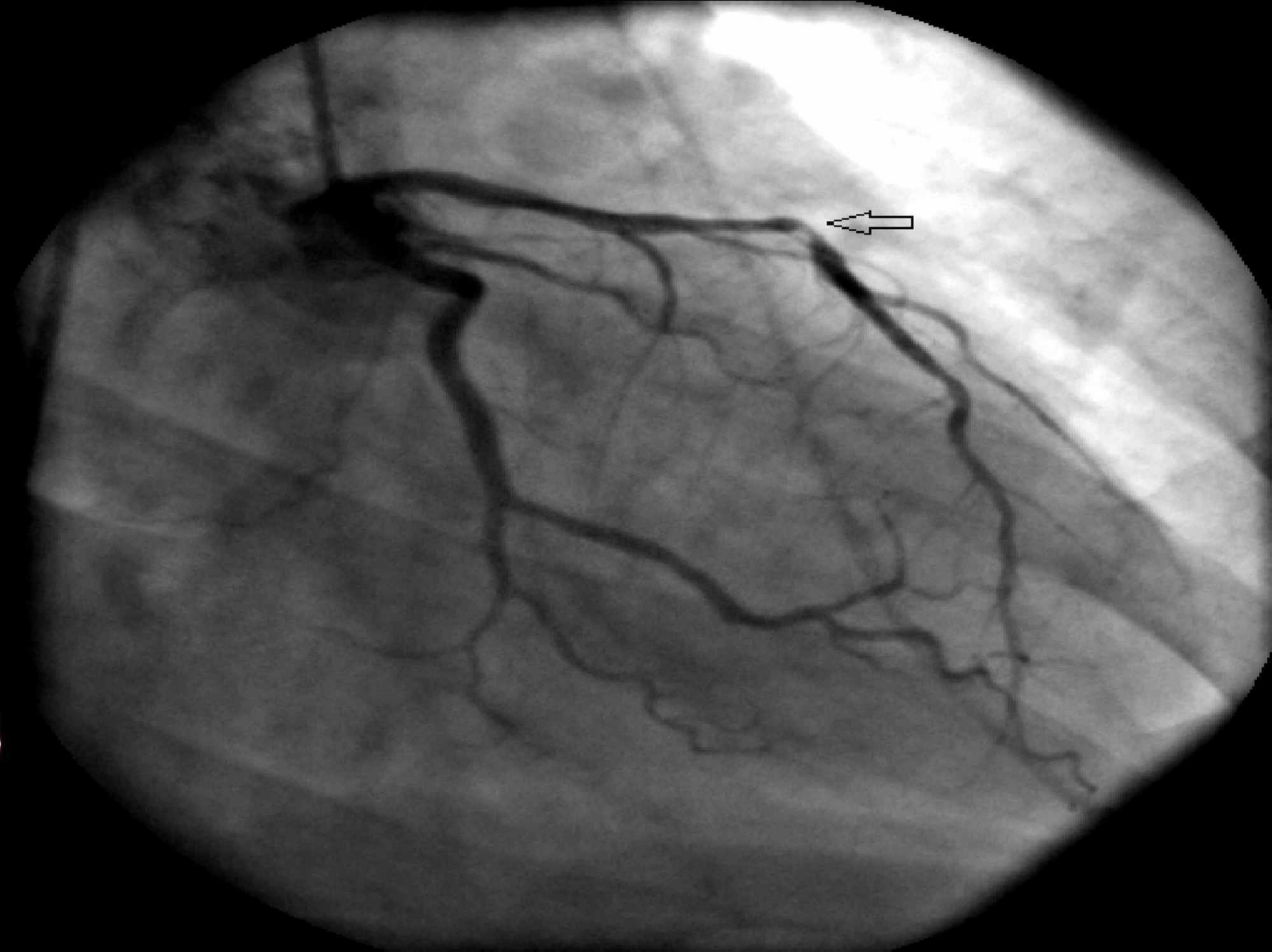
1. Right anterior-oblique caudal view on coronary angiogram (two years later) Occluded left anterior descending artery (open arrow)

Two years later, while on optimal therapy for the secondary prevention of CAD, and despite normal CRP, she presented with an acute anterior wall myocardial infarction. Cardiac catheterization revealed an acutely occluded mid-left anterior descending artery managed with a drug-eluting stent this time (Figure 2). There was no evidence of diffuse coronary artery disease on the coronary angiogram and no coronary calcifications on the chest CT scan. CRP at the time was normal while on treatment with methotrexate and folic acid. This prompted a workup to rule out alternate causes of thrombophilia that may have contributed to left anterior descending artery thrombosis despite apparently successful efforts to minimize modifiable known-risk. Compound heterozygous MTHFR mutations (C677T and A1298C) with high-normal plasma homocysteine (15 µmol/L) were detected.

Pertinent investigations

1. Plasma homocysteine 15 µmol/L (5-15 µmol/L)

2. Folic acid 12.3 ng/ml (>3.0 ng/ml)

3. Erythrocyte sedimentation rate (ESR) 20 mm/hr (0-20 mm/hr)

4. CRP 1.2 mg/dl (0-4.9mg/dl)

5. HbA1c 8.5%

6. Partial thromboplastin time (PTT)-lupus anticoagulant 37.4s (0-50s)

7. Dilute Russell's viper venom time (dRVVT) 32.9s (0-55.0s)

8. B2-glycoprotein immunoglobulin (Ig) A/G/M 2.9 GPI

9. Glucose-6-phosphate dehydrogenase (G6PD), quantitative 225 (146-376)

10. Anti-double-stranded deoxyribonucleic acid (anti-dsDNA) 4 IU/ml (0-9IU/ml)

11. C3 11.7mg/dl (90-180 mg/dl); C4 33mg/dl (9-36 mg/dl)

12. Methylenetetrahydrofolate reductase (MTHFR) gene PCR amplification followed by restriction analysis positive for C677T and A1298C mutations

Outcome and follow-up

Optimal medical therapy for CAD included lifelong dual anti-platelet therapy with aspirin and clopidogrel in addition to atorvastatin and metoprolol. Behcet’s disease continues to be in clinical remission with methotrexate and off-label use of Apremilast. Folic acid therapy was continued.

Eighteen months later, the patient reported new-onset exertional dyspnea and underwent another cardiac catheterization, which revealed patent left anterior descending and circumflex stents with no evidence of obstructive CAD. During the subsequent 18 months, the patient did not experience any acute cardiac events. Despite aggressive measures, HbA1c continues to be high (8.0%).

## Discussion

Diabetes mellitus is a well-established risk factor for atherosclerosis and considered to be a coronary artery disease equivalent [7], which commonly presents with multivessel obstructive atherosclerotic lesions in diabetic patients [1]. Furthermore, juvenile diabetes accelerates early atherosclerotic lesions and presents with very severe CAD [8]. Despite the history of Type I diabetes, the patient described in our case had no evidence of diffuse coronary artery disease on two coronary angiograms and no coronary calcifications on the chest CT scan.

Systemic inflammation in Behcet’s disease leading to coronary arteritis is an independent risk factor for coronary atherosclerosis. It is believed that subclinical inflammation may cause the progression of atherosclerosis in Behcet’s disease otherwise in remission when immunosuppressive therapy is discontinued [9]. In the presented case, systemic inflammation is assumed to be quiescent based on normal inflammatory markers (ESR: 20 mm/hr and CRP 1.2 mg/dl).

Various thrombophilic factors have been evaluated in patients with Behcet’s disease and thrombotic events [10], one of which is methylenetetrahydrofolate reductase (MTHFR) polymorphism. MTHFR removes a methyl group converting 5,10-MTHF to 5-MTHF. The removed methyl group converts homocysteine to methionine. The activity of MTHFR in patients with compound heterozygous mutation (C677T and A1298C) is 50%-60% control activity, which is lower as compared to isolated heterozygous C677T mutation [11].

MTHFR mutations appear to be associated with elevated cardiovascular risk in specific populations with systemic inflammatory disorders like lupus and rheumatoid arthritis (RA) [5,12-13], independent of homocysteine levels. Studies on the association of heterozygous MTHFR mutations (C677T and A1298C) and CAD suggest that the T- and C-alleles may increase the risk of atherosclerosis [14-16]. In RA patients, C-allele carriers with the A1298C mutation and T-allele carriers with C677T polymorphism manifested an increased prevalence of atherosclerosis and a higher risk of five and 10-year cardiovascular events [5,13]. In patients with type 2 diabetes, heterozygous C677T mutations have been linked to a higher risk of stroke, which cannot be attributed to elevated homocysteine levels alone [17].

The synergistic effect of compound heterozygous MTHFR mutations on the risk of coronary artery disease in patients with Behcet’s disease and diabetes is unknown.

We hypothesize that the recurrent thrombotic events in this diabetic female, who did not have diffuse obstructive coronary disease, are likely due to the synergistic pro-thrombotic effects of compound heterozygous MTHFR mutations and Behcet’s disease. This hypothesis is favored by the control of recurrent coronary thrombosis with long-term dual anti-platelet and immunosuppressive and folic acid therapy aiming at reducing systemic inflammation. It is further supported by the absence of progression of atherosclerosis despite disappointingly suboptimal glycemic control.

Further studies are needed to determine the best management of patients with MTHFR mutations in the background of Behcet’s disease. Meanwhile, in this vulnerable subset of patients, it is prudent to employ a comprehensive approach to the cardiovascular risk reduction, including avoidance of smoking, lipid-lowering, and aggressive immunosuppressive therapy, with periodic assessment of CRP and homocysteine levels. In cases of thrombotic events, the decision to use long-term dual antiplatelet therapy should be accompanied by thoughtful consideration of the balance between the increased risk of bleeding in these patients and the benefits of antithrombotic therapy.

## Conclusions

Despite a history of diabetes, recurrent acute thrombotic coronary events in the young may be multifactorial. In Behcet’s disease, particularly in the presence of additional risk factors like diabetes and dyslipidemia, the treatment goal should be the normalization of inflammatory parameters. Consider compound heterozygous MTHFR mutations in patients with systemic autoimmune disease and recurrent thrombotic events despite quiescent inflammatory markers. The increased risk is not solely from hyperhomocysteinemia. Further studies may help elucidate the influence of compound heterozygous MTHFR mutations in patients with Behcet’s disease and recurrent coronary events. To conclude, despite the increased bleeding risk associated with Behcet's disease, drug-eluting stents for PCI and the long-term continuation of dual antiplatelet therapy may be preferred if additional cardiovascular risk factors are present.
